# Emerging diagnostic and therapeutic opportunities in food allergy

**DOI:** 10.3389/fimmu.2025.1715845

**Published:** 2026-01-22

**Authors:** Lukas Michaja Balsiger, Oliver M. Würgler, Hugh A. Sampson, Alexander Eggel

**Affiliations:** 1 Department for BioMedical Research, University of Bern, Bern, Switzerland; 2 Translational Research for Gastrointestinal Diseases (TARGID), KU Leuven, Leuven, Belgium; 3 Department of Rheumatology and Immunology, University Hospital Bern, Bern, Switzerland; 4 Seren & John Liew Division of Pediatric Allergy/Immunology, Icahn School of Medicine at Mount Sinai, Jaffe Food Allergy Institute, New York, NY, United States

**Keywords:** anti-IgE, basophils, bead-based epitope assay, diagnosis, food allergy, IgE, mast cell activation test, mast cells

## Abstract

Food allergy is characterized by acute onset of symptoms affecting multiple organ systems following the ingestion of otherwise innocuous food antigens. The prevalence of this potentially life-threatening disease is globally increasing and poses a significant burden on society and healthcare systems. In this review, we summarize the pathophysiology of food allergy focusing on pre-clinical evidence of how oral tolerance is broken and the role of systemic and mucosal immunoglobulin E (IgE) in food allergy. Diagnosing food allergy is complex and requires a multimodal approach. Importantly, the diagnostic accuracy of currently available tests is variable, and *in vivo* testing runs the risk of inducing anaphylaxis in patients. We summarize established diagnostic modalities and provide an overview of novel approaches that are currently in development to improve diagnostic accuracy while minimizing discomfort and risk of anaphylaxis. Finally, we comment on available treatment modalities and provide an outlook of new therapeutic options in clinical trials or under development.

## 1 Introduction

Characterized by the potential to induce life-threatening anaphylactic reactions, food allergies (FA) significantly impact quality of life and impose restrictions on daily activities, social engagement, and dietary habits. Most recent epidemiological data estimate the prevalence of food allergy between 3-10% in the United States and 1-9% in Europe (1). Although these numbers vary based across geographical regions and on the methodology used to assess the prevalence of food allergy, the overall prevalence of FA has substantially increased worldwide in the past years, both in pediatric and adult populations (1–3). It is difficult to ascertain true allergies from epidemiological data as there is a notable discrepancy between self-reported allergies and diagnosed FAs (4). However, robust clinical data such as hospital admissions due to food-related anaphylaxis clearly substantiate a continuous rise in FA over the past decades (1, 5).

Several epidemiological studies have shown an increased risk of developing FA in children with atopic skin conditions, such as atopic dermatitis (6, 7). These findings correlate with the concept of the “atopic march”, suggesting that epithelial barrier breakdown and inflammation in the skin create an environment for development of other allergic conditions (8). In this review, we will highlight recently discovered mechanisms of oral tolerance breakdown leading to production of food-specific immunoglobulin E (IgE) and development of FA. We will further discuss current and emerging diagnostic and therapeutic possibilities with a focus on the detection and targeting of food-specific IgE.

## 2 Pathophysiology of food allergies

### 2.1 Breakdown of oral tolerance and allergic sensitization

**Figure 1:**
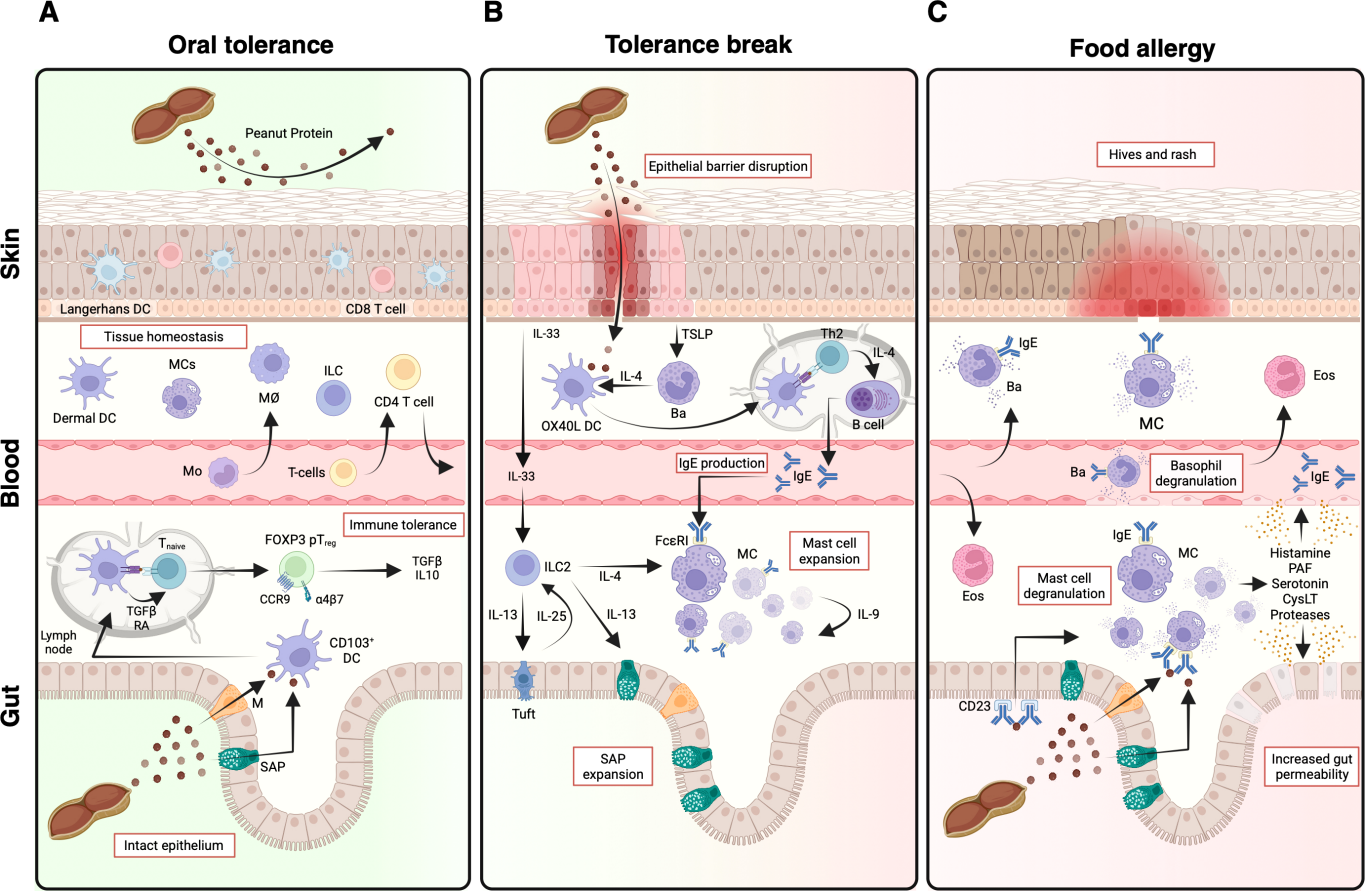
From oral tolerance to food allergy. **(A)** An intact skin barrier prevents the exposure to environmental food proteins with various immune cell types contributing to tissue homeostasis. Ingested food proteins (like peanut) are transported across the gut epithelium by specialized cells, taken up by antigen-presenting cells (APCs) such as CD103^+^ dendritic cells (DCs) and transported to mesenteric lymph nodes (mLNs). Here, antigen-specific FOXP3-expressing peripheral T regulatory cells (pTregs) are induced through the interaction with TGFβ- and retinoic acid (RA)-secreting APCs. pTregs then migrate back to the intestinal lamina propria in a CCR9- and integrin α4β7-dependent manner where they provide immune tolerance and mediate the functional unresponsiveness to innocuous foreign antigens. **(B)** In individuals with a disrupted skin epithelial barrier (e.g. atopic dermatitis) various immunological processes can lead to a break in tolerance and allergic sensitization. Many of these processes are started by damaged keratinocytes secreting the alarmins TSLP and IL-33. TSLP leads to the recruitment and expansion of IL-4 secreting basophils in the skin. Together with OX40L^+^ DCs, that have taken up antigen through the disrupted barrier, TSLP elicited basophils cooperatively induce T helper 2 (Th2) cell polarization in skin draining lymph nodes. Here, antigen-specific Th2 cells act on B cells through type 2 cytokines including IL-4 and IL-13 to induce IgE production. Food allergen-specific IgE then binds to the high-affinity IgE receptor FcϵRI on mast cells (MCs) leading to their sensitization. IL-33 promotes the expansion and activation of innate lymphoid cells type 2 (ILC2s) which is further amplified by a feedforward loop involving ILC2-derived IL-13 and Tuft cell-derived IL-25. Activated ILC2s secrete IL-13 promoting the formation of secretory cell antigen passages (SAPs) and IL-4, leading to the expansion of local MC populations. **(C)** Further oral exposure to the eliciting food antigen leads to transport of the allergen across the epithelium by various mechanisms where it crosslinks receptor-bound IgE on MC in the skin and gut and basophils in the blood resulting in immediate release of pre-formed mediators causing clinical symptoms ranging from hives and rashes in the skin and diarrhea or to systemic life-threatening anaphylaxis. DC dendritic cell; MC mast cell Mo macrophage; M m-cell; TSLP thymic stromal lymphopoietin; SAP secretory cell antigen passage; Eos eosinophil; PAF platelet activating factor, CysLT cysteinyl leukotrienes. *Created in BioRender. Eggel*, **(A)***(2025)*https://BioRender.com/0xc43l0.

The functional unresponsiveness to orally administered innocuous foreign antigens including dietary proteins termed oral tolerance is a key feature of the intestinal immune system (9) (reviewed in great detail elsewhere (10, 11)). Even though the crucial involvement of T cells has repeatedly been shown in experimental animal models (12) as well as in humans (13), the underlying mechanisms and involved cell types have only recently been elucidated (**Figure 1A**). In a pivotal study Coombes and colleagues demonstrated that antigen-specific peripheral FOXP3^+^ T regulatory cells (pT_regs_) are induced in mesenteric lymph nodes (mLN) after oral administration of antigen through the action of TGFβ and retinoic acid (RA) secreting CD103^+^ dendritic cells (DCs) (14). pTregs then migrate to the intestinal lamina propria in a CCR9 and α4β7 integrin- dependent manner, where they suppress adverse reactions and provide immune tolerance (15). These findings have been replicated numerous times and pTregs are now recognized as key drivers of oral tolerance (**Figure 1A**) (16, 17). Recently, another T cell subset contributing to oral tolerance termed lineage-negative CD4^+^ T helper cells (T_H_^lin-^) has been identified. These are hyporesponsive in their primed state, lack inflammatory functions and can differentiate into pTregs in an IL-2-dependent manner upon further dietary antigen exposure (18). Besides pTregs, induction of oral tolerance requires professional antigen presenting cells (APCs), which can drive tolerogenic responses. Several studies have suggested a key role for classical DCs (cDCs) in the induction of oral tolerance (14, 19). However, recently a novel proximity-based labelling approach enabled the identification of dietary antigen-presenting migratory cDC1 and Rorγt^+^ APCs as the main drivers of pTreg generation (20). Shortly after, a subset of Thetis cells, a lineage of Rorγt^+^ APCs, were found to induce food-specific pTregs during weaning in mice promoting oral tolerance in this critical early phase of life (21). Additional recent work has reinforced the importance of non-ILC3 Rorγt^+^ APCs in the induction of tolerogenic pTregs in later phases of life (22–25) although a consensus still has to be reached on the nomenclature and phenotypic description of the different subsets identified (26, 27).

In the past 20 years, the newly gained knowledge and interest in oral tolerance was applied in breakthrough studies challenging the long held dogma of early life avoidance to prevent allergy by showing that early introduction of peanuts into the diet of infants decreased the prevalence and risk to develop peanut allergy (28, 29). This was first demonstrated in an observational study comparing Jewish children in the UK (where infants avoided peanuts) and Israel (where peanut consumption is high in infants) (28) and later validated by different randomized control trials such as the Learning Early about Peanut Allergy (LEAP) trial (29, 30).

While oral uptake of food leads to tolerance, there is increasing evidence from experimental mouse models and human studies that exposure to dietary antigens via other surfaces such as the skin or the airways can lead to allergic sensitization (**Figure 1B**), which has been termed the dual allergen exposure hypothesis (31, 32). Early evidence came from the observation of the typical progression of allergic diseases starting in infancy with atopic dermatitis (AD) followed by IgE-mediated FA and later progression to asthma and allergic rhinitis, termed the atopic march, with type 2 inflammation as an underlying factor for all of them (33). By the early 2000s the Avon Longitudinal Study of Parents and Children (ALSPAC) study identified oozing and crusting skin rash as an independent risk factor for the development of peanut allergy and the authors hypothesized that sensitization might have occurred due to the application of creams containing peanut oil to the disrupted and inflamed skin epithelium (**Figure 1B**) (34). A range of studies have now confirmed AD as a risk factor for allergy against various foods in different cohorts (35–37) and interventional studies further strengthened the causal link between AD and FA by showing that early aggressive topical corticosteroid therapy in infants with AD reduced incident FA at two years of age, an effect that was enhanced with an earlier start of treatment after AD onset (38, 39). Similarly, treating patients suffering from atopic dermatitis with the anti-IL-4Rα antibody dupilumab has been associated with a decreased development of various allergies including FAs, although not reaching statistical significance (40).

To elucidate the underlying cellular mechanisms linking skin damage to FA, a lot of work has focused on experimental mouse models. The first evidence for epicutaneous sensitization in mice was published in the early 2000s where an OVA-impregnated patch applied to dorsal shaved skin for one week induced OVA-specific IgE and caused anaphylaxis upon oral challenge with OVA. Both of these effects were shown to be IL-4 dependent as they could be reversed in anti-IL-4 antibody treated mice (41). Later studies used tape stripping of the skin to break the epithelial barrier onto which the antigen could be added allowing for a more physiologically short exposure of antigen. Early experiments using this model showed the induction of a food allergic phenotype by breaking down oral tolerance or preventing its induction (42). Later it was shown that tape stripping and epicutaneous sensitization with OVA, but not oral immunization with adjuvants, was sufficient to drive expansion of mast cells (MCs) in the small intestine (SI), increase serum IL-4 levels, and induce allergen-specific IgE production and anaphylaxis upon oral challenge (43). The underlying mechanism governing the expansion of MCs after skin injury was later elucidated in a groundbreaking study by Leyva-Castillo and colleagues which demonstrated the presence of a skin-gut axis (**Figure 1B**). Here, tape stripping-induced skin damage led to increased serum levels of the alarmin IL-33 secreted by keratinocytes in the skin, which together with tuft cell-derived IL-25 promoted the expansion and activation of ILC2 cells in the gut. At the same time, ILC2-derived IL-13 drove tuft cell expansion resulting in an intestinal ILC2-tuft cell feedforward loop (**Figure 1B**). ILC2-derived IL-4 and IL-13 then directly acted on intestinal mast cells driving their proliferation (44). Moreover, ILC2-derived IL-13 was found to be crucial to increase the formation of secretory epithelial cell antigen passages, termed SAPs, in the small intestine (SI) demonstrating a possible mechanism for the transport of food antigen across the SI to induce IgE-mediated anaphylaxis (**Figure 1B**) (45, 46). Other groups have shown a key role for TSLP in the development of allergen specific IgE especially in the early phases of an AD model (47). In their animal model of AD, TSLP knockout mice did not develop intestinal MC expansion suggesting a key role for TSLP in the skin-gut axis. The exact interaction of alarmins released by keratinocytes and intestinal MCs has not been fully elucidated but is likely complex and may differ between animal models of AD. It seems that both IL-33 and TSLP play key roles in the induction of intestinal MC expansion in AD.

Additionally, IL-25 has been implicated in the pathogenesis of FA in dedicator of cytokenesis 8 (DOCK8) deficient patients, who are highly susceptible to FA (48). Using DOCK8-deficient mice, the authors showed that DOCK8 is crucial for regular Th17 and Treg function, as DOCK8 deficiency led to reduced IL-17 levels and dysbiosis driving increased IL-25 production by tuft cells. This promoted Th2-derived IL-4 production that prompted expansion of mucosal mast cells (MMCs) and oral anaphylaxis, and DOCK8-deficient Tregs were unable to suppress these processes (48). Expansion of MCs in the SI was also observed in a small cohort of human AD patients. However, it is important to note that four out of eight patients suffered from gastrointestinal Th2 diseases (2 eosinophilic esophagitis, 2 FA) and thus did not represent a *bona fide* cohort of pure AD (44). Data to corroborate the link of atopic dermatitis and intestinal mast cell infiltration in human are scarce with older studies showing increased densities of unspecified IgE positive cells in the small intestine of children with AD (49, 50). Larger scale studies are required to corroborate these findings to reinforce a potential link with the increased risk for FAs in these patients. Another commonly used mouse model for AD and FA research includes the cutaneous application of the vitamin D3 analog MC903, which induces TLSP expression in keratinocytes resulting in an AD-like phenotype (51). TSLP contributes to the induction of a T_H_2 response driving allergic inflammation and potentially FA by acting on several cell types including DCs, ILC2s, CD4^+^ T cells and basophils (52). Notably, TSLP induces expansion of phenotypically and functionally different basophils subsets compared to IL-3, which have been termed TSLP-elicited basophils (53). In FA models combining MC903-induced skin disruption and epicutaneous sensitization with OVA, it has been found that TSLP-elicited basophils expand in the skin where they promote antigen-specific T_H_2 responses and increased antigen-specific serum IgE levels (**Figure 1C**) leading to the development of FA (54). They do so by providing an early source of IL-4 and through reciprocal cell-cell interactions with DCs leading to OX40L upregulation on DCs to cooperatively induce T_H_2 polarization in skin draining lymph nodes (55–57). T_H_2 cells then act as key orchestrators of the food allergic response by secreting type-2 cytokines including IL-4 and IL-13 that drive class switch recombination from IgG to IgE in B cells leading to increased antigen-specific serum IgE levels (58).

### 2.2 Role of IgE in food allergy

In 1967, IgE was the last human antibody isotype to be discovered (59). Similar to IgG antibodies, IgE consists of two identical heavy and light chains. However, unlike IgG, the IgE heavy chain contains an additional Ig-domain and lacks the flexible hinge region (60). While IgE is the least abundant antibody in human serum with a relatively short half-life of roughly 2–3 days in circulation (61, 62), it plays a pivotal role in the development and manifestation of FAs.

Soluble IgE binds with high-affinity to its primary receptor FcϵRI expressed on allergic effector cells including mast cells and basophils in different tissues such as the skin, lung and gastrointestinal tract as well as systemically in the blood (**Figure 1C**) (63). Upon contact with the sensitized allergen, receptor-bound IgE is cross-linked leading to immediate cell activation and the release of pre-formed mediators from mast cells or basophils, as well as *de novo* formation of pro-inflammatory mediators and cytokines. While mast cells were implicated in promoting homeostatic intestinal barrier function (64), their activation-induced release of mediators has been reported to increase epithelial permeability and thereby to increase luminal antigen-uptake (65, 66). Furthermore, released mediators directly trigger the clinical symptoms of FA ranging from diarrhea or hives to life threatening anaphylaxis when the reaction occurs systemically (63, 67, 68) and are crucial in the regulation of food avoidance behavior (69, 70). In the following sections, we will focus on the role of systemically measured serum IgE versus local IgE in the mucosa of the luminal gastrointestinal (GI) tract.

### 2.3 Systemic IgE versus local IgE

#### 2.3.1 Blood

Animal models using passive sensitization suggest that systemic allergen-specific IgE is sufficient to trigger anaphylactic shock upon oral exposure and systemic absorption of allergen (71, 72). However, many FA animal models, regardless of active or passive sensitization, use either intravenous or intraperitoneal routes of allergen challenge, which is not reflective of orally induced FA (73, 74). In humans, transfusing plasma from donors with food specific-IgE was shown to induce transient *de novo* sensitization in previously unsensitized recipients (75). Importantly, two case reports suggest that this passive sensitization to foods following transfusion was indeed sufficient to induce anaphylaxis following the ingestion of respective foods (76, 77). However, these case reports must be considered with caution. While one case was a child that had undergone radio-chemotherapy and multiple transfusions, the other case was an elderly woman. In both cases the patients had at least some level of altered immune function at the time of passive sensitization.

Systemic IgE is pivotal in the development of FAs as demonstrated in the LEAP trial, where the development of peanut allergy was assessed in children followed until the age of five years (29). Children that developed FA during the study began developing high levels of peanut-specific IgE at 2.5 years of age, an observation that was not seen in tolerant children (78). Interestingly, early oral introduction of peanut did not prevent generation of IgE against whole peanut but rather prevented development of linear epitope-specific IgE and led to the expansion of protective allergen-specific IgG4 (78). Short-lived circulating IgE^+^ plasma cells (PCs) as well as longer-lived IgE^+^ PCs in the bone marrow likely represent the main sources of IgE measured in the serum. This is corroborated by the finding that the amount of circulating IgE^+^ PCs is correlated with serum IgE (79), both in food allergic individuals and mixed cohorts including healthy controls (80) and that eradication of IgE^+^ PCs significantly reduces IgE levels (81). While the presence of food specific serum IgE is associated with FAs, the positive and negative predictive values of specific serum IgE for a clinical reaction to double blind food exposure vary between cohorts and seem to vary between tested allergens (82–85). In a meta-analysis including data from 2831 patients, measuring food specific IgE resulted in a good sensitivity for most allergens, but specificity remained poor, again varying between different allergens (86). Conversely, in peanut allergy a study found good specificity and negative predictive values but poor sensitivity for peanut specific IgE depending on the cut-offs used (87).

Further evidence for the importance of systemic IgE has recently been shown by the therapeutic effect of omalizumab, a monoclonal anti-IgE antibody capable of binding and neutralizing free IgE in the serum (88). Omalizumab has shown efficacy in the treatment of FAs but in the limited number of studies that reported immunological readouts, serum sIgE did not differ in patients with omalizumab plus OIT versus patients with OIT only or with pre-omalizumab levels (89). More recently, treatment of patients with multiple FAs with omalizumab outperformed placebo (90). Therapeutic targeting of IgE is discussed in more detail in the dedicated therapy section of this review.

#### 2.3.2 Gastrointestinal mucosa

**Figure 2:**
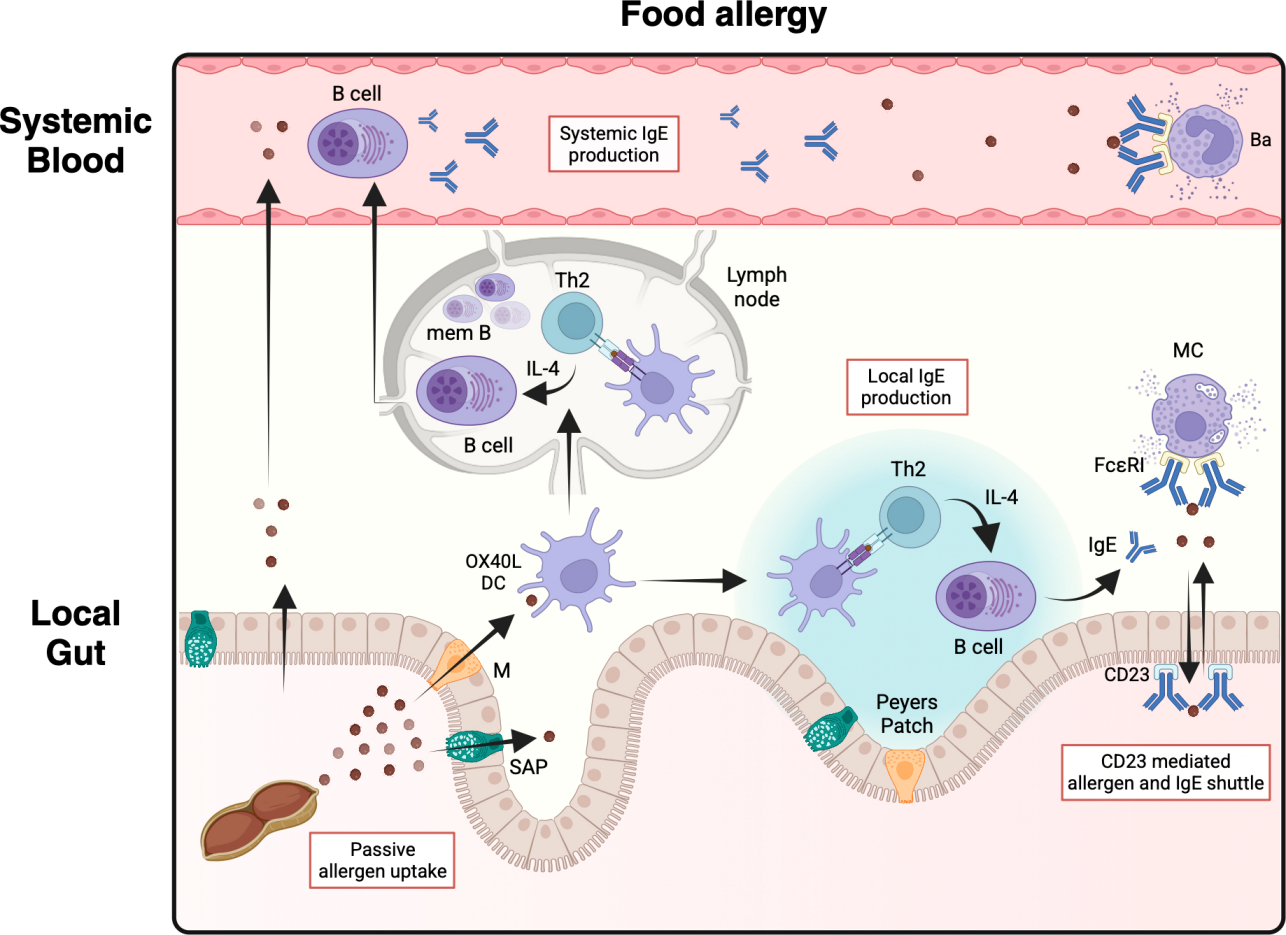
Systemic and local production of IgE. Switched plasma cells circulating in the blood stream cause systemic IgE production representing a main source of IgE in the serum. Circulating IgE leads to sensitization of effector cells. Locally in the intestinal mucosa, OX40L dendritic cells (DCs)- and T helper 2 (Th2) cell-mediated class switch recombination of B cells takes place in Peyer’s patches leading to local IgE production. Local IgE binds to the low-affinity IgE receptor CD23 on epithelial cells and to the high-affinity IgE receptor FcϵRI on resident mast cells (MCs). IgE reaches the intestinal lumen via basolateral CD23 mediated transcytosis. In turn, IgE-bound allergen complexes are transported into the lamina propria via apical CD23. Transported IgE:allergen complexes are able to directly lead to mast cell degranulation upon crosslinking. IgE Immunoglobulin E; Th2 T helper 2 cell; M M-cell; SAP Secretory cell antigen passage; Ba Basophil; mem B IgE memory B cell; FcϵRI high affinity IgE receptor. *Created in BioRender. Eggel, A. (2025)*https://BioRender.com/ib2i9uf.

Mechanistically, the presence of IgE in the gastrointestinal mucosa has been shown to enhance allergen uptake (91, 92). Importantly, in *ex vivo* experiments, IgE-mediated epithelial transcytosis via the low-affinity IgE receptor CD23 was necessary to induce allergic reactions (91). Similarly, *ex vivo* models have shown that CD23-mediated uptake of IgE:allergen complexes protected the allergen from degradation leading to rapid translocation of intact allergen (93). In human derived cell culture models, transcellular CD23-mediated uptake of IgE:allergen complexes were protected from enzymatic degradation. Importantly, the intact IgE:allergen complexes were able to activate unsensitized mast cells (**Figure 2**) (94).

Evidence for the mechanistic role of local IgE remains sparse. While the presence of local IgE in FA might be a contributing factor, it is not sufficient for the development of FA. Notably, recent animal models of post-infection and stress-related irritable bowel syndrome have demonstrated local IgE production in the colon and small bowel of animals without systemic manifestations of FA upon food exposure (95, 96).

The presence of antibodies against food allergens in the stool in the absence of systemic sensitization has been reported in a case series of children suffering from chronic diarrhea as early as 1969 (97). Upon exclusion of the culprit foods from their diet, these patients showed clinical improvement. However, the involvement of IgE antibodies in this observation remains unclear. Although this patient cohort likely reflected a heterogenous group rather than *bona fide* FA, it is still suggestive of a link between antibodies against foods in the intestinal lumen and clinical symptoms (97). Similarly, local production of IgE in the gastrointestinal mucosa was described in two children with cow's milk allergy with increased numbers of small intestinal IgE plasma cells after cow milk exposure (98). In the same line, a case report found increased IgE positive cells in the jejunum of an allergic individual compared to controls and compared to patients with eosinophilic infiltrates alone (99).

A study analyzing multiple gastrointestinal tissues and circulating plasma cells has shown evidence suggestive of IgE isotype class switch taking place locally in the GI tract in peanut allergic individuals (**Figure 2**) (100). In this group of patients, IgE^+^ plasma cells were most prevalent in the stomach and in the duodenum. These data suggest that the GI mucosa might present a relevant reservoir of IgE producing cells in individuals with FA (100). Local IgE has also been demonstrated throughout the luminal GI tract in healthy volunteers and IgE^+^ cells made up for around 2% of all immunoglobulin containing cells (101). Interestingly, the authors also reported the presence of IgE in small intestinal fluid in almost all samples and found no differences between health and parasitosis (101). More recent data confirmed the presence of IgE in the luminal GI tract with higher values measured in more proximal segments and significantly higher values observed in food allergic individuals (102). There is limited evidence showing a discrepancy between systemic and local reactions to foods. In pioneering work, Bischoff and colleagues injected foods in the submucosa of patients with suspected FAs and observed wheal and flare reactions in more than three quarter of patients (103). This observed mucosal reaction was linked to local mast cell activation. While the reactions corresponded to the clinical history of patients, there was no association with skin prick test results nor serum IgE values. Unfortunately, the local IgE values were not reported in that study (103). Another method of sampling the luminal gastrointestinal tract is through stool samples. There is limited evidence suggesting the presence of IgE in the stool of allergic individuals but not healthy controls (104). In a small case series of pediatric patients undergoing oral food challenge, food specific IgE was indeed measurable in the stool (94). More recently, IgE was demonstrated in pediatric patients with peanut allergy in which fecal IgE was correlated with abdominal pain suggesting that luminal IgE might be reflective of certain aspects of the clinical phenotype (105).

## 3 Diagnosis of food allergies

**Figure 3:**
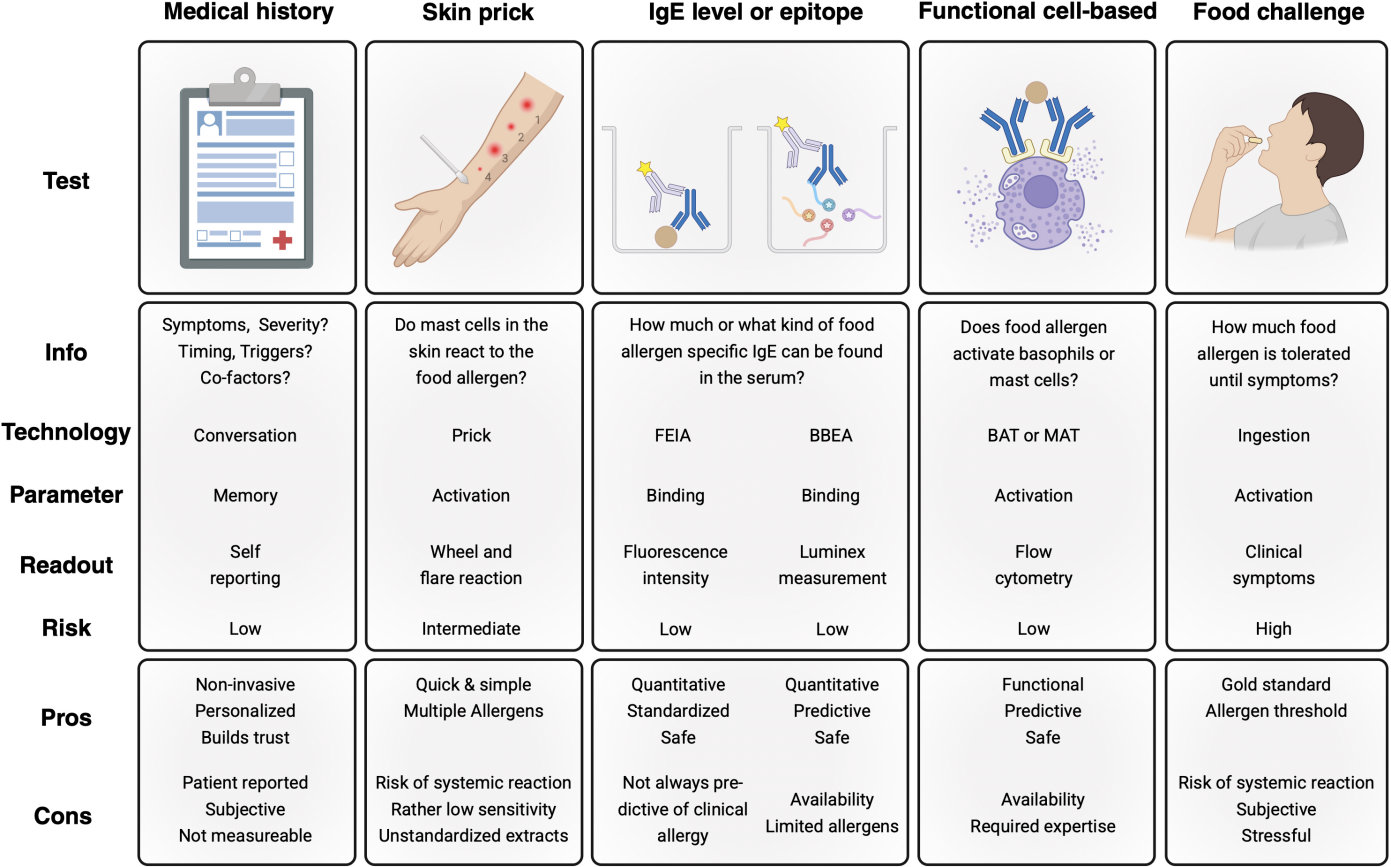
Modalities used to diagnose food allergy. Diagnosis of food allergy is a multimodal approach that should be performed by experienced physicians. The basis of all diagnostic approaches is medical history compatible with food allergy. Confirmatory testing is performed to demonstrate food-specific immunoglobulin (Ig) E sensitization. Skin prick and serum measurements of IgE levels are well established and are part of the diagnostic workup suggested by international guidelines. While FEIA is widely available, BBEA is currently only approved for the diagnosis of peanut allergy in the United States. BAT and food challenge can be considered in expert centers if the previous testing is equivocal. MAT has not yet gained regulatory approval as a diagnostic tool but holds promise to complement the diagnostic armamentarium in the future. Risk of the individual tests refers to the likelihood of inducing a systemic allergic reaction (106, 107). FEIA Fluorescence enzyme immunoassay; BBEA Bead-based epitope assay; BAT Basophil activation test; MAT Mast cell activation test. *Created in BioRender. Eggel, A. (2025)*https://biorender.com/27b29ae.

The accurate diagnosis of FAs is a complex multistep procedure with different available tests that should be performed by specialized allergologists (**Figure 3**). Although there is a limited amount of high-quality studies assessing the performance of clinical history, it represents an important basis for diagnosing FAs (108). Medical history generally revolves around the questioning of symptom patterns including the type of exposure, the presumed allergen, the timing and reproducibility of symptoms, and the environment (i.e. newly introduced to the diet or staple food) (109). IgE-mediated FAs typically cause symptoms affecting multiple organ systems including cutaneous (generalized flushing, urticaria, angioedema), abdominal (cramps, nausea, vomiting, diarrhea), respiratory (stridor, wheezing) manifestations, and potentially anaphylactic shock. Symptoms typically occur within thirty minutes to two hours of exposure, although they might occur later in the specific case of alpha-gal syndrome (108). Importantly, in the case of IgE-mediated FAs, symptoms are generally reproducible upon re-exposure (109). In some cases, cofactors such as exercise, NSAIDs and/or alcohol in addition to ingestion of the food are necessary to provoke an allergic reaction (110).

A clinical history compatible with FA alone is not sufficient to make the diagnosis but a history not suggestive of classical FA will significantly decrease the pretest probability of confirmatory testing. If the history is suggestive, confirmatory testing is required to document IgE sensitization. In this case, skin prick testing (SPT) and/or serum specific IgE measurement are indicated as next steps in the workup of suspected FA.

### 3.1 Skin prick testing

SPT is performed by administering allergen onto the skin and pricking the skin with a lancet (111). By disrupting the epithelial barrier, the allergen reaches the subepithelium, where it binds to mast cell sensitized with specific IgE, triggering their degranulation. Release of preformed mediators such as histamine leads to local wheal and flare reactions (112). SPT are considered positive if an allergen leads to the formation of a cutaneous wheal within 15 minutes of performing the skin prick. The size of wheal to be considered positive varies between different allergens (113). Due to the iatrogenic injury caused by the prick and highly variable interindividual skin sensitivity, a positive and negative control always have to be included to correctly identify positive wheal and flare reactions (114).

The sensitivity and specificity of SPTs vary between allergens and even between various preparations of allergens (e.g. cooked vs raw egg white) (115). Depending on the allergen and the population assessed, SPTs have a specificity for FA of 66% (raw egg white; cut-off > 6mm) to 92% (cashew; cut-off > 5mm) and sensitivity of 47% (soy; cut-off > 3mm) to 93% (cashew; cut-off > 5mm) (115). The allergens to be tested should be informed by the medical history and can be tailored to the patients’ individual situation. Indiscriminate testing for food allergens is discouraged (108). Drawbacks of SPT are the requirement of trained staff (including emergency equipment for the treatment of rare anaphylactic reactions) and potential interference of medications, e.g. antihistamines, with test interpretation (114). Furthermore, results vary between individuals requiring a rigorously standardized approach to performance and interpretation within each institution (114). Depending on the number of tested allergens, SPTs might be perceived as unpleasant and stressful by the patients, which often are children. Advantages include the immediately available results and the fact that results are directly seen by the patients themselves (116).

### 3.2 Allergen-specific IgE measurements

Food specific IgE can be measured in serum using various techniques, the most common of which is the ImmunoCAP^®^ platform allowing the detection and quantification of IgE specific to a wide variety of food allergens (117). In principle, a solid phase loaded with allergen extracts or component proteins is used to bind allergen specific antibodies from a serum sample. Bound IgE is detected through enzyme labelled anti-human IgE. The platform has shown high sensitivity, a long linear range of quantification and does not cross-react with human IgG. Additionally, many allergen extracts and allergen molecules are now commercially available (117).

While historically a threshold value of >0.35 kU_A_/L sIgE is commonly used to define sensitization, newer assay technologies use lower thresholds of 0.1 kU_A_/L. However, the sole presence of sIgE does not indicate functional activity or predict clinical reactivity. Some studies suggest that more than 80% of individuals classified as sensitized based on such assays do not experience allergic symptoms when challenged with allergen (118). This commonly observed phenomenon is now widely known under the term “sensitized (but) tolerant”. As with SPT, cut-off values and performance characteristics of sIgE measurements vary between different food allergens and components. The sensitivity of sIgE varies from 40% (baked egg white; cut-off: 8 kU_A_/L) to 96% (shrimp; cut-off: 1.2 kU_A_/L) and sensitivity varies from 63% (shrimp; cut-off: 1.2 kU_A_/L) to 94% (baked egg white; cut-off: 8 kU_A_/L) (115). Additionally, there is a high false positive rate when total IgE are very high (119) and an increased risk of false negatives when total IgE levels are undetectable.

Measuring serum specific IgE has the advantage that it is highly standardized from the allergen panel to quantification of results and that it does not carry any risk of inducing anaphylaxis in patients (117). Furthermore, it is easy to test specific allergen components, leading to increased diagnostic resolution and sometimes even better sensitivity and specificity such as for example in the case of whole peanut extract versus Ara h 6 testing (115). Another potential advantage of measuring specific allergen components is the detection of molecules that frequently cause cross reactions. For example, it has been shown that the major birch allergen Bet v 1 is a root cause for cross-reactive FA with inhalant allergy due to structural protein homologies in Western Europe (120). This is important as extracts used for SPT often contain varying amounts of specific allergen components. The prick solutions are poorly standardized and might even lose their allergenic potential following prolonged storage (120).

### 3.3 Bead-based epitope assay

**Figure 4:**
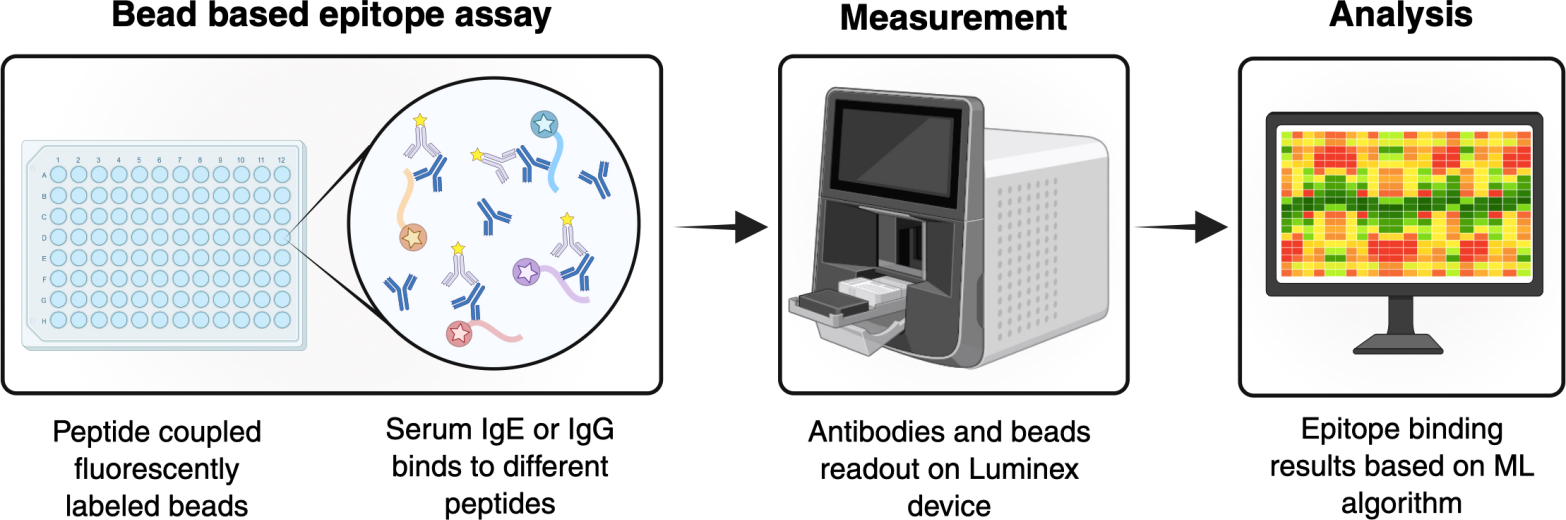
Schematic workflow of bead-based epitope assay (BBEA): Fluorescently labelled beads coupled to known allergenic epitopes are incubated with patient serum. Using secondary antibodies specific for IgE or IgG, the serum content of epitope specific antibodies is measured using a Luminex device. The analysis is performed using complex bioinformatics including machine learning. IgE Immunoglobulin E; IgG Immunoglobulin G; ML Machine learning. *Created in BioRender. Eggel, A. (2025)*https://BioRender.com/ww1kc1d.

The amino acid sequences of most major food allergen component proteins have been delineated and by synthesizing overlapping peptides (generally 15- to 20-mers) representing the full length of these component proteins, linear allergenic epitopes have been identified using various microarray platforms (121). The BBEA is a high-throughput assay in which individual peptides are coupled to unique LumAvidin^®^ beads (**Figure 4**). A mix of up to 100 different peptide-containing beads is incubated with patient serum or plasma in 96-well plates. Secondary PE-labeled antibody is added, e.g. anti-IgE, anti-IgG4, etc. and the signal is quantified as a Median Fluorescence Intensity (MFI) using a Luminex multiplex cytometer. Results of the BBEA have been shown to be highly reproducible, with a greater sensitivity compared with those of peptide microarray assays (122).

Using bioinformatic techniques, including machine learning, IgE antibodies to specific “informative” epitopes have been found to be more predictive of clinical reactivity than currently available tests. The BBEA for peanut has been validated and approved for use in the United States (123), and similar assays for milk, egg and wheat are being validated (121). The BBEA is being developed to address more than a patient’s binary allergic classification. For example, an algorithm for the peanut BBEA has been developed that provides information on a patient’s degree of clinical sensitivity, i.e. how much peanut protein they likely can tolerate before developing an allergic reaction (124). Earlier studies identified epitope-specific IgE antibodies profiles that predict which “high-risk” infants are likely to develop persistent peanut allergy (125), which patients undergoing oral immunotherapy are likely to achieve sustained unresponsiveness vs. desensitization (126), and the potential severity of an allergic reaction following accidental peanut ingestion (127).

### 3.4 Functional cell-based tests (BAT & MAT)

**Figure 5:**
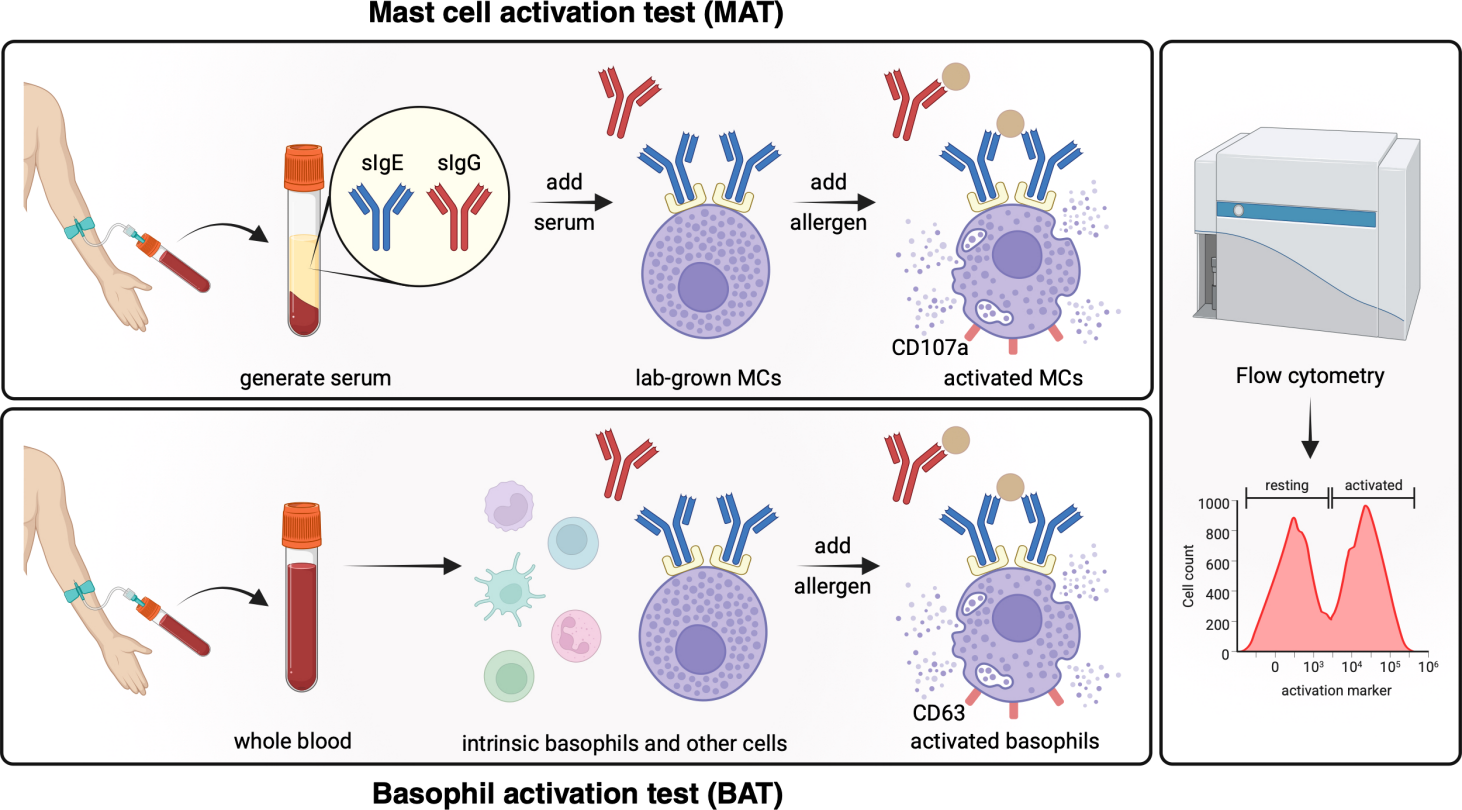
Ex vivo effector cell activation tests. For the mast cell activation test (MAT), lab-grown mast cells are sensitized with patient serum. Upon allergen exposure, FcϵRI crosslinking of sensitized mast cells leads to their degranulation. Degranulation leads to the exposure of proteins such as CD107a on the cell surface, which can be quantified using flow cytometry. Patient derived basophils in whole blood are used for the basophil activation test (BAT). Adding allergens leads to FcϵRI crosslinking of *in vivo* sensitized basophils. Basophil degranulation leads to CD63 exposure on the cell surface which again can be measured using flow cytometry. sIgE serum immunoglobulin E; sIgG serum immunoglobulin G; MCs mast cells. *Created in BioRender. Eggel, A. (2025)*https://BioRender.com/0dssfu9.

To capture the functional relevance of sIgE and to identify real cases of clinically relevant sensitization, a variety of *ex vivo* cell-based assays have been developed. These aim to replicate the allergic reaction in a safe and controllable environment *in vitro* by quantifying the activation of allergic effector cells in response to culprit allergens. However, sourcing primary cells directly from the patients is associated with major logistical and biological challenges. Mast cells predominantly reside in peripheral tissues and can only be accessed through invasive biopsies, which yield limited cell numbers insufficient for routine testing (128, 129). Consequently, alternative cell sources and assay formats have been explored to enable functional allergy diagnostics at scale (**Figure 5**).

The basophil activation test (BAT) is currently the most established functional *ex vivo* assay. In case first-line testing (i.e. SPT or serum IgE) is equivocal or in case results are contradictory, functional testing using BAT has been suggested as a second line diagnostic test according to most current guidelines (108). The development of BAT was enabled by the identification of CD63 (LAMP3) as a marker of basophil activation that becomes exposed on the cell surface upon stimulation with anti-IgE or fMLP (130). Flow cytometry-based detection of CD63 and later CD203c (E-NPP3) (131) on basophils in whole blood allowed for assay performance without the need for prior cell purification. Numerous academic laboratories and commercial entities utilize the BAT to assess whether patient basophils respond to allergen stimulation in an IgE-dependent manner. The diagnostic accuracy of BAT for common food allergens including peanut, tree nuts, milk, egg, wheat, and sesame has been assessed in multiple studies (132). While generally high sensitivity and specificity are reported (115), it is important to note that the performance metrics vary depending on the allergen source and protocol used. While the BAT has recently been incorporated into updated EAACI guidelines for FA diagnosis (108), its broader implementation has been limited due to logistical challenges, high inter-donor variability as well as critical protocol differences between different laboratories. Even after efforts to standardize methodologies, substantial inter-laboratory variability remains, with reported standard deviations ranging from 16.2% to 49.2% (133). Moreover, many studies exclude individuals whose basophils fail to respond to positive controls such as anti-IgE or anti-FcϵRI stimulation. These so-called “basophil non-responders” represent 2–17% of FA cohorts and up to 20% in other allergies (132, 134–136), suggesting that previously reported performances may be overly optimistic.

The mast cell activation test (MAT) has recently gained traction as an alternative functional assay format (137). MAT employs laboratory-grown mast cells such as Hoxb8-MCs, LAD2 cells, or CD34^+^ blood stem cell-derived human mast cells (hMCs), which express high levels of FcϵRI. These cells can be passively sensitized with patient serum to capture IgE, followed by stimulation with allergens and flow cytometric detection of activation markers such as CD63 or CD107a. Also, for the MAT, multiple protocols using different mast cell types are utilized across academic centers (132). However, since the same cell batch can be used within one experiment assessing many patient samples, inter-individual variability is significantly reduced. Studies assessing the diagnostic accuracy of MAT have thus far been limited to peanut allergy (138–140). Among the cellular sources tested, hMCs and Hoxb8-MCs showed the highest diagnostic performance, with sensitivities and specificities well beyond 90%. Notably, two studies included both “sensitized (but) tolerant” individuals and samples from basophil non-responders, with MAT successfully distinguishing allergic from non-allergic subjects in these difficult subgroups. Also, the Hoxb8-MAT has recently shown promising results in the longitudinal treatment follow-up of peanut allergic patients undergoing oral desensitization.

Each functional cell-based *ex vivo* assay has its unique advantages and limitations (137). BAT, which is based on whole blood, captures the individual’s *in vivo* immune context and intrinsic basophil reactivity, which may be influenced by disease state, allergen exposure history or treatment (141). However, the prevalence of non-responders (142), the variability in responsiveness (134, 143), and time-sensitive sample requirements pose considerable practical constraints. Conversely, MAT offers robust assay stability and scalability, as serum can be frozen and mast cells are grown in controlled laboratory conditions, enabling high-throughput analyses suitable for clinical trials for example. Yet this comes at the cost of omitting intrinsic patient-specific cellular information, which seems to be less relevant for diagnosis than the humoral factors present in the blood, as demonstrated by high diagnostic accuracy in clinical utility studies using MAT (140). Ongoing studies, optimization efforts and commercial development strategies will soon reveal how these new functional *ex vivo* tests will be adopted by food allergy patients and healthcare providers.

### 3.5 Oral food challenge

For diagnostic workup in everyday clinical practice, the oral food challenge (OFC) represents the last recommended diagnostic approach in case previous diagnostic tests failed to provide a definitive diagnosis (108). In clinical studies, an OFC performed as a double-blind placebo-controlled food challenge (DBPCFC) is typically used as a surrogate marker to ensure assessment of relevant endpoints in a standardized manner and considered the gold standard in diagnosing FA. Typically, OFCs consist of exposure to increasing amounts of the food allergen in question at fixed time intervals. In the absence of clinical symptoms, the dose is increased, and the test is continued until the pre-defined maximal dose is achieved or until symptoms occur (144). Determining whether *de novo* occurring symptoms are indeed caused by allergic reactions is not always straight forward and requires clinical judgement by experienced providers and this is even more complex when testing pediatric patients (145). Often results are only reported as eliciting dose, which does not take into account change in symptoms, which may be clinically relevant. Moreover, data on the reproducibility of OFC reactions within individuals over time are limited. For cow's milk, 80% of patients undergoing blinded OFC reacted to the same dose upon re-exposure. The remaining patients showed clinical reactions following exposure to doses within plus or minus one dosing interval (146). Whether reproducibility of OFC reactions varies between allergens has not been assessed to the best of our knowledge.

While considered the gold standard, oral food challenges may also be false positive in the case of vocal cord dysfunction or severe aversion leading to vomiting. Rare cases of stridor as manifestation of Munchausen syndrome have also been reported and may lead to false positive results (145, 147, 148). False negative results may occur if concomitant medication has not been stopped masking the clinical manifestation of FA or if the titrated exposure leads to short-term specific oral tolerance induction as has been suggested by some authors (149). Importantly, conducting oral food challenges requires experienced providers in a specialized setting with appropriate resources to treat anaphylaxis and potentially anaphylactic shock. The risk of anaphylaxis is inherent to OFCs and thus a major drawback of this testing procedure, i.e. the induction of a potentially life-threatening situation for the patient (145).

## 4 Therapeutic approaches

**Figure 6:**
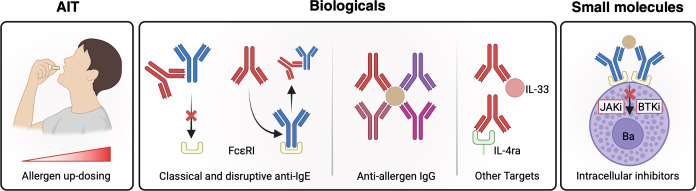
Therapeutic strategies for the treatment of food allergy. Allergen-specific immunotherapy (AIT) describes the (oral) exposure of allergens using increasing doses. Anti-IgE molecules bind free IgE and prevent effector cell sensitization. Disruptive anti-IgE molecules actively dissociate bound IgE from its receptor. Anti-allergen IgGs are allergen specific IgG antibodies that competitively bind allergen and thus prevent allergen-IgE interaction. Antibodies against IL-4ra and IL-33 inhibit the effect of Th2 cytokines with the aim of inhibiting the pathophysiological cascade leading to food allergy. Small molecules inhibit pathways downstream of FcϵRI crosslinking and have been proposed for the treatment of food allergy. FcϵRI high-affinity IgE receptor; IL-4ra Interleukin 4 receptor alpha-chain; IL-33 Interleukin 33; BTKi Bruton’s tyrosine kinase inhibitor; JAKi Janus kinase inhibitor. *Created in BioRender. Eggel, A. (2025)*https://BioRender.com/qxxg5hn.

Therapeutic options for FA aiming to reestablish tolerance remain limited, despite growing clinical interest. In the context of food−allergy treatment desensitization is increasingly recognized as a clinically meaningful endpoint. It is defined as a state in which the patient can tolerate a higher amount of allergen, while on active therapy, than prior to treatment without eliciting an allergic reaction. However, desensitization remains distinct from true remission or sustained unresponsiveness (SU). While desensitization depends on ongoing regular allergen exposure, SU refers to the capacity to tolerate the allergen even after a period of avoidance. Currently, only two therapeutic products have received regulatory approval: a GMP-grade, defatted peanut flour preparation (i.e. AR101, Palforzia^®^), approved in 2020, and the anti-IgE monoclonal antibody omalizumab (i.e. Xolair^®^), approved in 2024. To aid clinicians and general practitioners in managing IgE-mediated FAs, updated guidelines have been published by GAL2EN and EAACI (108, 150). In this section, we highlight the most extensively studied and promising emerging treatment strategies for food-allergic patients (**Figure 6**, **Table 1**).

**Table 1:** Advantages and limitations of different treatment approaches in food allergy.

Treatment approach	Advantages	Limitations	References
Allergen immunotherapy	Demonstrated efficacy of increasing clinical reactivity threshold Possibility for multi-allergen desensitization Multiple routes of administration (OIT, SLIT, EPITC, SCIT) possible	Risk of (severe) adverse events during updosing No validated biomarkers predicting long-term outcome No standardized treatment protocols and allergen formulations Limited durability of SU in most cases	(151, 153, 171, 193)
Vaccination	Favorable safety and tolerability with reduced risk of systemic reactions Various clinically proven vaccine platforms available Allows for rationale epitope design, potentially targeting multiple allergens	Limited clinical data, clear efficacy and durability have yet to be demonstrated	(174, 230, 231)
Anti-IgE (non-disruptive)	IgE is an optimal target due to its crucial role in FA and low serum concentration Few side effects and effective in patients with multiple FAs Could promote safety and efficacy of AIT when used in combination	Mainly manages symptoms, no evidence of long-term benefits such as SU Protection only from accidental allergen exposure. Avoidance is still advised Expensive and requires frequent injections	(89, 90, 232)
Anti-IgE (disruptive)	Same benefits as for non-disruptive anti-IgE biologics and additionally: Rapid active removal of IgE from effector cells. Longer-term allergic control due to FcϵRI downregulation on effector cells	Clinical studies demonstrating safety and efficacy are required	(88, 210, 233)
Anti-IL-33	Potential to modulate disease-driving type 2 inflammation Single dose sufficient to increase threshold of clinically tolerated allergen	Long-term benefits such as the durability of desensitization remain unclear	(220, 229)
Anti-IL-4R	Proven safety and efficacy in other atopic disorders Promote safety and efficacy of OIT when used in combination	Monotherapy not sufficient to induce effective clinical desensitization	(218, 219)
Passive immunization	Fast acting by direct neutralization of allergen Circumventing need for allergen exposure making it potentially safer than AIT	Long-term protection and disease modification unclear Monoclonal approaches not sufficient, need polyclonal anti-allergen IgG formulations	(223, 225)
Small molecules	Can be administered orally with rapid onset of action	Unwanted side effects due to off-target activity Unclear long-term safety and efficacy	(227, 228)

### 4.1 Food allergen immunotherapy (OIT, SLIT, EPIT)

Allergen-specific immunotherapy in FA (FA-AIT) has been most extensively studied for peanut allergies and to a lesser extent for cow’s milk and hen’s egg allergies (151). The procedure typically follows a structured protocol beginning with a dose escalation phase, during which the patient is exposed to very small amounts of the allergen. This is done under close medical supervision due to the risk of systemic reactions by the direct exposure to the treatment. If tolerated, the dose is gradually increased until a target maintenance dose is reached. Among the various strategies, oral immunotherapy (OIT) has emerged as the most extensively studied and clinically validated (152). While there is no standardized protocol with optimal duration of therapy, evidence from various clinical trials suggest a treatment window of 3–5 years (153). This long duration in conjunction with the remaining risk of systemic reaction leads to rather poor adherence to the therapy and a high rate of premature terminations, especially in clinical trials and academic centers with older patients with persistent food allergy and atopic comorbidities. In this context, alternative administration routes have been explored including sublingual (SLIT), and epicutaneous (EPIT) immunotherapy. SLIT delivers allergen often in glycerol solution to overcome taste aversion and offers a more favorable safety profile compared to OIT (152, 154). While SLIT provides a lower level of protection compared to OIT (i.e. lower threshold levels), studies in peanut allergy suggest that most individuals undergoing SLIT are likely to reach thresholds of 300mg (155) – typically associated with clinically relevant protection (156) - or higher (157, 158). EPIT, which is a novel, epidermal route of allergen delivery into intact skin using allergen patches, has also demonstrated efficacy in the EPITOPE trial (NCT03211247) (159), particularly in toddlers, and may be more acceptable in terms of adherence (152). EPIT leverages the immunological capacity of skin-resident dendritic cells, particularly epidermal Langerhans cells, which capture allergen and migrate to regional lymph nodes via lymphatics, facilitating T cell education while minimizing systemic exposure and thus the risk to induce anaphylaxis. The maintenance dose highly depends on the route of application and may range from hundreds of micrograms (EPIT) up to several grams (OIT) of allergen protein, which have to be administered daily. This maintenance phase is essential to sustain desensitization and promote more durable immunological changes (160). Various well controlled trials have consistently demonstrated that OIT can significantly increase the reactivity threshold for induction of clinical symptoms. While such treatments significantly decrease the risk of severe reactions upon accidental exposure, there is still limited evidence whether FA-AIT could be curative in the sense that the culprit food can be consumed in unrestricted amounts over prolonged periods of time after cessation of therapy. For example, in the PALISADE trial, two-thirds of participants receiving peanut OIT (e.g. Palforzia®) were able to tolerate a cumulative dose of >1 g of peanut protein after one year treatment (inclusion criterion: symptom onset at ≤100 mg) (161). In the IMPACT trial, 71% of participants could tolerate 5 g of peanut protein after 2.5 years of OIT treatment (inclusion criterion: symptom onset at <300 mg), suggesting a high rate of desensitization, which was mostly limited to the subgroup of younger infants with low specific IgE. However, much of the protective effect was lost in roughly two-thirds of study participants after 6 months of avoidance. Similarly, 85% could tolerate a cumulative dose of 4 g of peanut protein after 2 years of OIT treatment in the POISED trial, but this number dropped to 20% after 6 months cessation and 13% after cessation of the treatment for one year (162).

While standard in aeroallergen immunotherapy, subcutaneous immunotherapy approaches (SCIT) have been largely abandoned in FA due to a high rate of systemic reactions (163, 164). Newer strategies, however, involving hypoallergenic molecules (165–167) are being revisited via the subcutaneous route and hold promise for the future. Hypoallergens are structurally altered allergen proteins or peptides, which lose the capacity to degranulate allergic effector cells but maintain the ability to re-establish tolerance.

Food-allergic patients are often sensitized to more than one allergen (168). This so-called polysensitization leading to multi-FA complicates management. Therefore, studies of multi-allergen OIT have been started and shown that simultaneous desensitization to multiple foods is both feasible and efficacious. In one study, children with two or more FAs underwent multi-food OIT and achieved desensitization to a range of allergens with a safety profile comparable to single-food OIT (169–171). These findings challenge the long-held assumption that multi-food desensitization would pose excessive immunological or safety risks and open the door to broader, more personalized treatment approaches. Nonetheless, translating these findings into commercial therapies remains a major challenge due to regulatory hurdles, particularly those surrounding the characterization, standardization, and stability of multi-component biologic products.

Besides classical OIT, various other active immunization strategies have emerged. Different approaches of active immunization with food allergens to re-establish oral tolerance are currently under investigation. While classical allergen immunotherapy (AIT) is mostly based on the use of natural allergens, newer approaches rely on the application of recombinant wildtype allergens, hypoallergens as well as carrier-bound allergens or allergen peptides in conjunction with different adjuvants. Recently, favorable safety and tolerability data from two phase 1 clinical trials with either peanut peptides (172) or a peanut component (i.e. Ara h 2) coupled to a virus-like particle carrier have been communicated (173–176). Other approaches with hypoallergens are still in pre-clinical development phases (167, 177, 178). Ultimately, it will be interesting to learn which of these approaches will demonstrate the highest level of efficacy and whether active immunization strategies could even be combined in patients with severe allergies to multiple foods.

At the immunological level, FA-AIT induces a series of complex and time-dependent changes that ultimately lead to clinical tolerance or even sustained unresponsiveness in some cases (179). One of the earliest effects is a rapid functional desensitization of mast cells and basophils. This phenomenon, also known as “allergic effector cell anergy” occurs within days of initiating therapy and results in a decreased propensity of these cells to degranulate in response to allergen exposure (180). It happens even before significant reductions in allergen-specific IgE take place indicating that subthreshold piecemeal release of mediators could be involved, which depletes granule content and raises the activation threshold of these cells leading to their exhaustive state (i.e. anergy) (181). Another study has reported that early suppression of basophil activation might be dependent on rapid upregulation and activation of histamine receptor 2 (182). In FA, this immediate suppression of effector cell reactivity provides critical early protection during the escalation phases of OIT, where the risk of systemic reactions is highest. As treatment progresses, a more profound and durable immune reprogramming takes place. Central to this process is the induction and expansion of allergen-specific regulatory T cells (Tregs), including both natural FoxP3-expressing CD4+CD25+ cells and inducible IL-10-secreting Tr1 cells (183, 184). These Tregs play a pivotal role in suppressing the pathogenic Th2 responses that drive IgE-mediated allergy. They exert their effects through secretion of regulatory cytokines such as IL-10 and TGF-β, which not only dampen Th2 cytokine production, but also suppress the activity of dendritic cells, mast cells, basophils, and eosinophils (185, 186). In parallel the treatment leads to a gradual downregulation of Th2 cell differentiation, shifting the balance of CD4+ T cell responses away from a type 2 response and toward a more regulatory and Th1 phenotype (187). While allergen-specific IgE levels may transiently rise in response to continued allergen exposure, they typically decline over time with continued treatment (188). More importantly, there is a robust and sustained increase in allergen-specific IgG production. In atopic children in the LEAP trial, peanut-specific IgG1 was associated with allergy while IgG4 was the best biomarker for exposure (189). IgG4 is often viewed as a hallmark of successful immunotherapy. These IgG4 antibodies may act as “blocking antibodies,” competing with IgE for allergen binding and thereby preventing cross-linking of IgE on mast cells and basophils (190). They can further inhibit allergic effector cell activation directly at the cell surface via inhibitory FcγRIIb engagement (191, 192). Taken together, these coordinated immunological changes, ranging from early effector cell desensitization to long-term rebalancing of T and B cell responses, underlie the clinical efficacy of food allergen immunotherapy (63, 193). In some patients, particularly those who begin therapy at young age, these mechanisms may eventually support sustained unresponsiveness, however, in most cases, the persistence of clinical protection requires ongoing engagement of the immune system through continuous exposure.

A limitation in FA-AIT lies in the absence of validated biomarkers that reliably track clinical response or predict long-term outcomes. Standard tools such as skin prick testing, specific IgE measurement, and even allergen-specific IgG4 responses have shown limited correlation with clinical protection. Thus, there is increasing interest in functional assays that measure effector cell reactivity as surrogate markers of immune modulation. BAT has been extensively studied as a tool for monitoring the efficacy of allergen immunotherapy, including oral (OIT), sublingual (SLIT), and epicutaneous (EPIT) approaches for peanut, egg, and milk allergies (194). In peanut OIT, early and sustained reductions in basophil activation were associated with favorable clinical outcomes (195, 196), and low baseline activation levels predicted better long-term tolerance (195). Decreased basophil reactivity has also been observed following peanut SLIT (157, 158) and egg OIT (197, 198). However, reductions in basophil responsiveness may be transient (199), limiting their utility as durable biomarkers. At present, no studies have evaluated MAT for longitudinal monitoring of food allergen immunotherapy. Nonetheless, preliminary work using MAT in grass pollen SLIT suggests the potential for longitudinal monitoring of treatment efficacy (200).

### 4.2 Monoclonal anti-IgE antibodies

The central role of immunoglobulin E (IgE) in driving allergic diseases has provided a strong rationale for developing targeted therapies that disrupt IgE-mediated mechanisms. One significant advantage in targeting IgE lies in its relatively low serum concentration (~350 ng/mL) compared to other immunoglobulin isotypes such as IgG, which circulates at levels around 10 mg/mL. This allows for an efficient blocking approach that can neutralize IgE effectively using therapeutically achievable doses of anti-IgE agents without the need for unfeasibly high drug amounts. Over the past two decades, anti-IgE therapies have revolutionized the management of several allergic conditions. Numerous clinical investigations have shown efficacy of neutralizing circulating IgE in the treatment of allergic conditions. Despite the successful use of omalizumab, the first approved anti-IgE monoclonal antibody, limitations such as dosing restrictions and the frequency of injections have spurred efforts to develop next-generation anti-IgE agents with improved properties.

#### 4.2.1 Omalizumab

Omalizumab was the first monoclonal anti-IgE monoclonal antibody to be approved for clinical use, laying the foundation for targeting IgE in allergic diseases. Its development was guided by three essential functional principles: First, high-affinity binding to free IgE, which competes effectively with the high-affinity receptor FcϵRI; second, selective binding that spares IgE already complexed with FcϵRI, thus avoiding unintended receptor crosslinking and activation of effector cells; and third inhibition of the interaction between IgE and CD23 on B cells, which helps prevent antigen presentation and subsequent IgE production. Derived from the murine antibody MaE11 and subsequently humanized into rhuMAb-E25 to retain high affinity through minimal framework modifications (201), omalizumab has since been approved for the treatment of a variety of allergic disorders. Its primary mode of action involves neutralizing free IgE and reducing its interaction with effector cells, which is further complemented by downregulation of FcϵRI expression on basophils and dendritic cells. More recently, it has been reported that omalizumab even has the ability to actively accelerate IgE dissociation from FcϵRI at high concentrations and over a timeframe of several days (202).

Following the encouraging results from stage 1 of a phase 3 clinical trial (i.e. OUtMATCH study), the monoclonal anti-IgE antibody omalizumab was approved for the treatment of FA in 2024 (90). The study investigated omalizumab as monotherapy for individuals with multiple FAs, including peanut, milk, egg, cashew, walnut, wheat, and hazelnut. Participants were allergic to peanut and at least two other foods, with confirmed reactivity to small amounts (≤ 100 mg peanut protein and ≤ 300 mg for other foods) on baseline food challenges. The primary endpoint was the ability to consume ≥ 600 mg of peanut protein in a single dose without dose-limiting symptoms. The key secondary endpoints were the ability to consume ≥ 1000 mg of cashew, milk and/or egg, also without dose-limiting symptoms. 67% of participants in the omalizumab group tolerated the target dose of peanut (Milk: 66%; Egg: 67%; Cashew: 41%), compared with only 7% in the placebo group (Milk: 10%; Egg: 0%; Cashew: 3%). These findings provided strong evidence that omalizumab significantly raises the threshold of reactivity to multiple food allergens, offering broad protection that could reduce the risk of severe reactions to accidental exposures. Recently, first results from stage 2 of the OUtMATCH study have been communicated, in which omalizumab monotherapy showed superiority to oral immunotherapy (203). Interestingly, the differences were largely due to high rates of adverse events leading to study discontinuation in the OIT treatment group. Future stages of the trial will assess long-term benefits, including whether omalizumab leads to disease modification and tolerance, as well as how it compares to combination therapy.

#### 4.2.2 Ligelizumab

Ligelizumab is an alternative anti-IgE antibody, which binds to free IgE with approximately 88-fold higher affinity than omalizumab and is currently in clinical development (204). It targets a distinct epitope that spans across both Cϵ3 domains of the IgE Fc region, thereby providing superior steric hindrance to the IgE: FcϵRI interaction. However, this binding orientation also results in reduced efficacy at blocking interactions with CD23 compared to omalizumab and unlike omalizumab it does not actively dissociate IgE from FcϵRI.

Preclinical studies and early-phase clinical trials demonstrated that ligelizumab effectively suppresses circulating free IgE levels, reduces FcϵRI receptor expression on basophils, and significantly diminishes skin test responses to allergens (205). These promising results have generated optimism about its utility in FA, where reducing the sensitivity of mast cells to allergens could translate into higher reaction thresholds and potentially protect patients against accidental exposures (206). One large, multicenter trial in peanut allergy was initiated (NCT04984876). However, it was recently terminated, because an interim efficacy review suggested that the dosing regimen might require optimization to achieve the desired clinical effect. There seem to be plans to restart the trial with revised dosing strategies, and long-term extension studies are already underway to assess the chronic use of ligelizumab over periods of up to three years.

#### 4.2.3 Next generation anti-IgE

Next-generation anti-IgE molecules build on the proven success of omalizumab while seeking to overcome its clinical limitations by enhancing both the potency and kinetic activity of IgE neutralization. We have recently described the active removal of FcϵRI-bound IgE as a novel additional mode-of-action for anti-IgE molecules (88, 202, 207). While the disruptive capacity of omalizumab is weak and requires high concentrations or long treatment duration, new candidates including engineered antibodies as well as alternative scaffolds with improved efficacy to dissociate IgE from its high-affinity receptor, FcϵRI, on allergic effector cells have been developed. Some efforts have focused on modifying omalizumab itself to yield variants with enhanced disruptive potency. For instance, using yeast-display screens, clone C02 has been identified that not only exhibited higher affinity but also effectively desensitized human basophils rapidly *in vitro* (208). Engineering efforts further optimized this candidate by inserting flexible glycine linkers in the Fab-elbow region to produce the omalizumab variant C02-H2L2, markedly increasing its ability to strip IgE from FcϵRI. Separately, structure-guided mutations led to the development of FabXol3, an omalizumab derivative with slightly higher affinity and superior capacity to accelerate IgE dissociation from its receptor (209).

A particularly innovative strategy involves a new class of molecules known as disruptive IgE inhibitors. Unlike classical anti-IgE antibodies, these agents are specifically engineered to actively remove IgE from receptor complexes. One such approach employs designed ankyrin repeat proteins (DARPins). For example, DARPin E2_79 has been shown to accelerate the dissociation of preformed IgE: FcϵRI complexes (207). Building on this work, bispecific constructs such as bi53_79 (generated by genetic fusion of distinct DARPins) (202) and the IgG Fc-fusion hybrid KIH_E07_79 (engineered using knobs-in-hole technology) have been developed (210), combining the rapid disruptive capacity of DARPins with the favorable pharmacokinetic properties of conventional IgG antibodies. Pre-clinical studies with KIH_E07_79 revealed a rapid on-set of action leading to the desensitization of allergic mice with a single injection of the molecule in less than one day (210). This therapeutic approach holds great promise not only for the treatment of FAs but also for other allergic conditions, in which time is of essence and rapid intervention is required. While active desensitization of human basophils and dendritic cells with disruptive IgE inhibitors has been demonstrated *ex vivo*, the *in vivo* safety and efficacy of this strategy in humans remains to be investigated.

### 4.3 Combined biological plus AIT

Omalizumab has emerged as a valuable adjunct to AIT, particularly in the context of FA where safety concerns and high rates of adverse events often limit the broader application of AIT strategies. Omalizumab mitigates this risk by binding to free IgE and lowering its availability to trigger mast cell and basophil activation. Its use as pre-treatment or concurrent therapy with AIT has been extensively studied, beginning with trials in respiratory allergies and expanding into FA contexts (211–217).

In FA, omalizumab has consistently demonstrated the ability to improve the safety and efficacy of OIT. For example, in a randomized controlled trial of multi-food OIT, 83% of participants receiving omalizumab passed a 2-gram food challenge to two or more allergens after 36 weeks, compared to only 33% in the placebo arm (211). Importantly, this effect extended across allergens, enabling desensitization to multiple foods simultaneously, a significant advancement over traditional OIT protocols. In addition to improved safety and tolerability, omalizumab-treated individuals reached maintenance doses more quickly, with reduced rates of dose-limiting reactions during the critical up-dosing phase. Although sustained unresponsiveness (SU) remains a challenge and often requires continued maintenance dosing, omalizumab has also shown potential to support longer-term tolerance. In one study, participants who achieved maintenance were randomized to continue different maintenance doses (1g, 300mg, or 0mg), with those maintaining 300mg or 1g doses demonstrating superior outcomes compared to those who stopped therapy entirely (217). This suggests omalizumab may support a more flexible and personalized approach to long-term FA management. Overall, omalizumab’s integration with AIT represents a promising shift toward safer, more effective FA therapy, especially for individuals at high risk of severe reactions or with complex multi-allergen sensitivities. It is now FDA-approved as an adjunct to allergen avoidance in FA and is increasingly being used to support AIT in both research and clinical settings. While further work is needed to define optimal dosing, duration, and patient selection strategies, current evidence strongly supports the role of omalizumab in expanding the safety and utility of food allergen immunotherapy.

Recently, a phase II, multicenter, randomized, double-blind study was conducted with the anti-IL-4 receptor alpha chain monoclonal antibody dupilumab in conjunction with OIT (NCT03682770) in pediatric patients with confirmed peanut allergy (218). Dupilumab was added to the peanut allergen product AR101 during the up-dosing and maintenance phases. Results demonstrated that patients receiving dupilumab with OIT had a 20.2% higher rate of desensitization, as measured by successful completion of a 2044 mg cumulative peanut protein food challenge after up-dosing, compared to OIT alone. Although this benefit was partially lost when dupilumab was discontinued, continuous treatment further improved outcomes during maintenance and follow-up. Dupilumab was well tolerated, reduced peanut allergy-related symptoms and gastrointestinal side effects, and led to reductions in total and peanut-specific IgE levels without increasing serious adverse events. These findings provided first evidence for added benefit of dupilumab co-application to increase safety and efficacy of OIT in pediatric food allergy.

### 4.4 Other approaches

#### 4.4.1 Monoclonal antibodies

A phase 2 clinical trial (NCT03793608) has been performed to evaluate the efficacy of dupilumab as a monotherapy in patients with IgE‐mediated peanut allergy (219). The primary endpoint was defined as the proportion of participants who safely passed a double-blind, placebo-controlled food challenge (DBPCFC) with a cumulative dose of at least 444 mg of peanut protein at week 24 (inclusion criterion: dose-limiting symptoms ≤100 mg peanut protein). Dupilumab monotherapy did not effectively induce clinical desensitization in most subjects. Only 8.3% of the 24 enrolled participants met the primary endpoint, while 41.7% of participants had to use adrenaline as a rescue medicine to treat adverse events upon OFC.

A single center proof-of-concept phase 2a clinical trial in peanut allergy has been performed with the monoclonal anti-IL-33 antibody etokimab (NCT02920021). Participants needed to have a clinical diagnosis of peanut allergy confirmed by OFC. On day 1, the participants received one intravenous dose of etokimab or placebo. Then they were re-challenged by OFCs on days 15 and 45. 73% and 57% of the etokimab-treated participants passed the defined OFC outcome on day 15 or 45, respectively (tolerating a cumulative 275 mg of peanut protein) compared with 0% in the placebo group (220). The study’s results suggest that a single dose of etokimab can significantly increase the threshold of peanut protein tolerated during an oral food challenge in peanut-allergic adults. These effects appear to be mediated by a reduction in key Th2 cytokines and a decrease in peanut-specific IgE levels. The favorable safety profile and the reduction in atopy-related events support the potential of IL-33 blockade as a therapeutic strategy for FA. However, given the small sample size and short follow-up duration, larger and longer-term studies will be needed to confirm these findings and to establish the durability of desensitization.

#### 4.4.2 Passive immunization approaches

Passive immunization strategies are emerging as a promising approach for the treatment and prevention of FAs. These strategies involve the administration of allergen-specific IgG antibodies, which can neutralize allergens and prevent IgE-mediated allergic reactions without the need for traditional allergen exposure inherent in AIT. Several studies have demonstrated that passive transfer of allergen-specific IgG can effectively inhibit the binding of allergens to IgE, thereby blocking the activation of mast cells and basophils. For instance, in murine models of peanut and fish allergies (221–224), passive immunization with allergen-specific IgG antibodies led to a marked reduction in allergic symptoms and inflammatory responses. Advancements in molecular immunology have facilitated the identification and cloning of human B cells that produce allergen-specific IgG or IgE antibodies. By isolating these B cells from allergic patients, researchers have been able to generate monoclonal IgG antibodies targeting specific epitopes of major food allergens, such as Ara h 2 in peanuts (223, 225). If used in the right polyclonal combination, some of these antibodies have demonstrated the ability to neutralize allergens effectively and prevent IgE-mediated activation of effector cells *in vitro* and *in vivo* in mouse models of peanut allergy. These allergen-specific IgGs have been shown to additionally act through the inhibitory FcγRIIb receptor (226). Collectively, these findings underscore the potential of passive immunization with allergen-specific IgG antibodies as a therapeutic modality for FAs. By circumventing the need for allergen exposure, this approach offers a safer and potentially more effective alternative to traditional AIT, with the added benefit of providing immediate protection against allergic reactions. However, successful inhibition of the allergen often requires the simultaneous blocking of multiple allergen epitopes indicating that monoclonal approaches will not be sufficient. Another question that remains to be answered is how long the protective effect of such a passive immunization will last and whether it has disease-modifying potential.

#### 4.4.3 Small-molecule inhibitors

Small-molecule inhibitors targeting the intracellular kinases such as JAK1 (e.g. abrocitinib) and BTK (e.g. acalabrutinib, remibrutinib), which are involved in cytokine signaling or associated with FcϵRI activation pathways that lead to mast cell degranulation, show promising early results for the treatment of FA. Acalabrutinib, in particular, has demonstrated increases in the tolerated threshold of consumed peanut in a short-term phase 2 clinical trial (NCT05038904), suggesting a rapid onset of action (227). The phase 2 study with remibrutinib has recently been completed (NCT05432388), but to date results are still pending. Ongoing trials with oral JAK inhibitors are primarily investigating biomarker changes and safety at this stage (NCT05069831) (228). Many small-molecule inhibitors that are now being investigated for treatment of allergies, were previously approved for therapeutic use in B-cell malignancies or allergic conditions such as atopic dermatitis. While these molecules have the advantage of oral administration, they might have a disadvantage in the specific targeting of allergic effector cells, which might be associated with unwanted side-effects via off-target activities. Whether JAK or BTK inhibitors are suitable to be used as standalone treatments or in combination with AIT protocols for the treatment of FAs remains to be further evaluated.

## 5 Discussion and outlook

Over the past decades, our understanding of the immunological mechanisms underlying FA has grown significantly. The central role of IgE in mediating hypersensitivity responses has provided a conceptual and therapeutic framework for both diagnosis and intervention. However, the rapid rise in FA prevalence, particularly in industrialized nations, underscores the complex interplay between genetic, immunological, and environmental factors, including skin barrier integrity, microbiome alterations, and dietary exposures. Key insights into oral tolerance, particularly the role of pTregs and antigen-presenting cells in the gut, have helped elucidate why and how tolerance is lost, while highlighting potential preventive and therapeutic avenues.

Diagnostics have evolved beyond the traditional measurement of allergen-specific IgE in serum, with functional cell-based tests like the BAT and MAT potentially offering a more robust and reliable correlation with clinical reactivity. While the OFC still remains the gold-standard, these up-coming *in vitro* tests might represent a promising and safe alternative in the near future.

Therapeutically, food allergen-specific immunotherapy has shown efficacy in increasing clinical allergen tolerance thresholds, particularly through oral, sublingual, and epicutaneous routes. The approval of Palforzia® for peanut allergy and positive outcomes in multi-allergen protocols mark significant clinical milestones. However, concerns around long-term adherence, risk of adverse reactions, and sustained unresponsiveness have limited its widespread adoption. Adjunctive strategies, particularly with biologics like omalizumab, have demonstrated clear benefits in improving the safety and tolerability of AIT, especially in multi-food allergic patients. The integration of omalizumab into AIT protocols is supported by growing clinical evidence and regulatory approvals.

Next-generation biologics and novel therapeutic platforms are currently being explored to expand the efficacy and accessibility of FA treatments. These include higher-affinity anti-IgE molecules, disruptive IgE inhibitors that actively remove IgE from effector cells, and bispecific antibodies targeting key cytokines and alarmins such as IL-33 and TSLP (229). Passive immunization with allergen-specific IgG antibodies represents another interesting frontier that could provide immediate and safe protection without the need for allergen exposure. Preclinical and early clinical evidence indicates that these IgGs can prevent IgE-mediated activation, however, more clinical data is needed to confirm these findings in human clinical trials.

Small-molecule inhibitors, particularly JAK1 and BTK inhibitors, are also emerging as promising tools. These agents, previously developed for other immune and neoplastic conditions, show potential for rapid desensitization and suppression of effector cell activation. Acalabrutinib, for instance, significantly raised reaction thresholds in a short-term trial, illustrating the feasibility of oral, short-course interventions. As these molecules are integrated into treatment paradigms, their long-term safety, specificity, and role in disease modification will require careful evaluation.

Moving forward, a major challenge will be the identification and validation of biomarkers that reliably predict therapeutic outcomes and guide individualized treatment. Integration of systems biology approaches, including multi-omics and machine learning, will likely be necessary to parse the heterogeneity of FA and treatment responses. Moreover, with the advent of combined and sequential therapies, there is a growing need to understand how these modalities interact to either potentiate or inhibit immunological tolerance.

In conclusion, FA research is entering a transformative era with multiple promising therapies on the horizon. The shift from avoidance to active disease modulation, particularly through immune engineering, biologics, and precision diagnostics, provides new hope for patients. However, translating these advances into universally accessible and safe treatments will require continued clinical innovation, robust regulatory pathways, and thoughtful integration into public health systems.
